# Influence of Menstrual Cycle Phases on Neural Excitability in the Primary Somatosensory Cortex and Ankle Joint Position Sense

**DOI:** 10.1089/whr.2020.0061

**Published:** 2020-06-16

**Authors:** Koyuki Ikarashi, Kaho Iguchi, Yudai Yamazaki, Koya Yamashiro, Yasuhiro Baba, Daisuke Sato

**Affiliations:** ^1^ Field of Health and Sports, Graduate School of Niigata University of Health and Welfare, Niigata, Niigata, Japan.; ^2^ Institute for Human Movement and Medical Sciences, Niigata University of Health and Welfare, Niigata, Niigata, Japan.; ^3^ Department of Health and Sports, Niigata University of Health and Welfare, Niigata, Niigata, Japan.

**Keywords:** menstrual cycle, primary sensory cortex, ankle, joint position sense

## Abstract

***Introduction:*** Ankle sprain (AS) is one of the most common injuries among women engaged in competitive sports and recreational activities. Many studies have shown that several factors contributing to AS are influenced by the menstrual cycle. Despite the finding that abnormal joint position sense (JPS) is one of the major risk factors of AS, the alteration of the JPS throughout the menstrual cycle and its associated neural mechanisms remain unclear.

***Objective:*** This study aimed to examine whether the menstrual cycle phases affect neural excitability in the primary somatosensory cortex (S1) and JPS.

***Methods:*** Fourteen right-footed women participated in this study. Somatosensory-evoked potential and paired-pulse inhibition (PPI) were measured to assess S1 excitatory and inhibitory functions. Ankle JPS was measured using an active joint position matching method. Menstrual syndrome was evaluated using the menstrual distress questionnaire. All assessments were conducted in the follicular, ovulatory, and luteal phases.

***Results:*** The two main findings of this study were as follows: First, PPI decreased in the ovulatory phase than in the follicular phase. This may have been the reason for estrogen altering the neural inhibition and facilitation balance throughout the menstrual cycle. Second, JPS was not changed during the menstrual cycle.

***Conclusion:*** In conclusion, phases of the menstrual cycle affect the neural excitability in S1 as shown by the decreased PPI in the ovulatory phase, and the ankle JPS was unchanged throughout the menstrual cycle.

## Introduction

Ankle sprain (AS) is one of the most common injuries in competitive sports and recreational activities. According to a previous study, 10%–30% of all athletic injuries are ankle injuries, and in many sports, AS accounts for ≥70% of all reported ankle injuries.^1^ Moreover, >40% of AS recur, and recurrent AS can lead to chronic ankle instability and ankle osteoarthritis.^2–4^ Risk factors reported for AS include anatomic natures,^5^ lower isokinetic strength,^6^ flexibility,^7^ joint position sense (JPS),^8,9^ muscle reaction time,^10^ postural stability,^11^ previous AS,^12,13^ and body mass index.^14,15^

According to a recent meta-analysis, women are at a higher risk of AS than men because of recurrent fluctuation in sex steroids by the menstrual cycle.^16^ Many studies have shown that several factors contributing to AS are influenced by the menstrual cycle.^17–19^ Although a meta-analysis revealed that abnormal JPS is one of the major risk factors of AS,^15^ the mechanism of JPS alteration throughout the menstrual cycle is still unclear. The methodology for assessing JPS is divided into two types of tasks, namely, passive and active joint position matching methods. These two methods differed in the presence or absence of an active motion when participants matched a given joint position. Especially, active JPS methods reflect the processing of not only external sensory feedback but also sensorimotor integration.^20^ A case study in Japan showed that passive ankle JPS was unaltered throughout the menstrual cycle in female volleyball players^21^; based on its findings, this study focused on active ankle JPS.

Previous studies showed that knee JPS is altered throughout the menstrual cycle, which would be attributed to proprioceptive and psychological functions. For example, Aydoğ et al.^22^ reported that proprioceptive deficits induced worse knee JPS during menstruation. In addition, proprioception can be influenced by emotional and environmental conditions, and knee JPS alteration may be caused by behavioral and emotional changes in the early phase of menstruation.^23^ Moreover, they speculated that the involvement of cortical activity in the alteration of JPS, with reference to some reports,^24–26^ described the relationship between the menstrual cycle and evoked potential measurements. However, since these studies evaluated the cortical activity in the visual and auditory cortices but not in the somatosensory cortex (S1),^24–26^ somatosensory evoked potential (SEP) and paired-pulse inhibition (PPI), which reflected neural excitability and inhibitory functions of the primary S1, should be investigated.

To address the research gap, this study aimed to examine whether the phases of the menstrual cycle affects neural excitability in S1 and the ankle JPS. As previous studies reported that estrogen reduces gamma-aminobutyric acid (GABA)ergic inhibition,^27^ we hypothesized that PPI, which is modulated by GABAergic neurons,^28^ will decrease in the ovulatory phase when the estrogen concentration is high. In addition, as the sensorimotor integration during active joint position matching tasks involves activation of S1,^29^ we hypothesized that ankle JPS will be altered with accompanying changes in PPI.

## Materials and Methods

### Participants

Fourteen healthy women aged 19–22 years volunteered to participate in the study. The participants were required to have no history of neurological or psychiatric disorders, including premenstrual dysphoric disorder or any self-reported major menstrual cycle-related changes in mood. They were also required to not be taking any ongoing prescription medications or using hormonal forms of contraception.

The study protocol was approved by the ethics committee of Niigata University of Health and Welfare, Japan. All experiments conformed to the Declaration of Helsinki. The participants provided informed written consent before participation.

After recruitment, all participants were required to measure their basal body temperature every morning immediately after waking up. Then, two menstrual cycles, leading up to their first session, were monitored, allowing an accurate estimate of the menstrual cycle length. All participants exhibited normal and regular menstrual waking cycles (35.24 ± 13.60 days) for the 2 months preceding the study. Based on the average menstrual cycle length of 28 days, the follicular phase was defined as days 3–5, the ovulatory phase as days 13–15, and the luteal phase as days 18–24. Since several participants had considerably longer or shorter cycles than 28 days, all participants were instructed to measure and report their basal body temperature every morning until the final session to adjust the examination date of their ovulatory and luteal phases accordingly. In particular, measurements in the ovulatory and luteal phases were performed after confirming a temporary decrease and increase in the basal body temperature, respectively.

### Experimental protocol

**FIG. 1.**
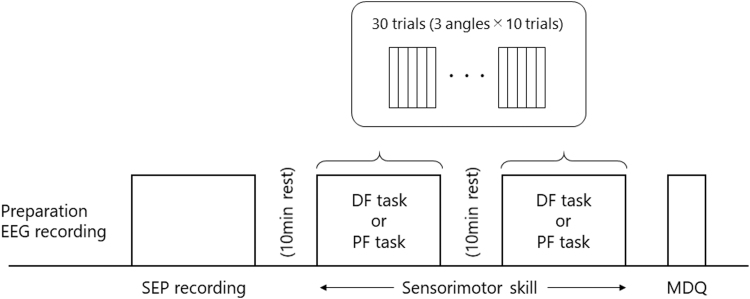
Experimental protocol.

Figure 1 shows the experimental protocol. All participants came to the laboratory on three instructed days corresponding to the follicular, ovulatory, and luteal phases. After preparation for electroencephalogram (EEG) recording, participants were instructed to sit on a reclining chair. SEP recording was conducted to evaluate PPI. After a 10-minute rest, two types of active joint position matching tasks were performed in random order: dorsal flexion (DF) and plantar flexion (PF) tasks (described as follows) with a 10-minute rest. After these neurophysiological and behavioral measurements, participants answered the menstrual distress questionnaire (MDQ) for evaluation of menstrual syndrome.

### Paired-pulse inhibition

**FIG. 2.**
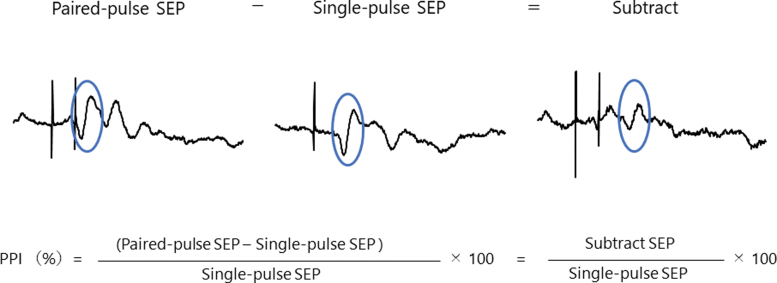
Representative data in single- and paired-pulse SEP waveforms and subtracted waveforms. Left panel shows single- and paired-pulse SEP waveforms. Right panel shows subtracted waveforms, which mean evoked responses for second stimulus only. Peak-to-peak amplitudes for N38-P45 component surrounded with circle were used to evaluate SEP and PPI because this component reflects neural activity in the primary somatosensory cortex. PPI, paired-pulse inhibition; SEP, somatosensory evoked potential.

PPI was measured to assess changes in S1 inhibitory function. Paired-pulse electrical stimulation (ES) of the common fibular nerve was applied with an interstimulus interval (ISI) of 5, 30, and 100 ms in combination with SEP recordings. The ISIs were determined based on the methods reported in previous studies.^30–34^ Participants were instructed to relax but stay awake without focusing attention on the stimulated leg. The right common fibular nerve was stimulated with an electrical stimulator through a surface bar electrode, with a cathode proximal. ES was delivered at 110% of the motor threshold and consisted of a square wave pulse of 0.2-ms duration. The active electrode was placed at Cz′ (located 2 cm posterior to Cz), the reference electrode was placed over Fz, and the ground electrode was placed on the right forearm using an earth band. Single-pulse and paired-pulse stimuli were randomly applied at a frequency of 2 Hz, which were controlled by the pulse control system (Pulse Timer II; Medical Try System, Tokyo, Japan). In each type of stimuli, 250 stimulation trials were performed. EEG signals were recorded at a sampling rate of 5000 Hz. Continuous EEG data were applied with a band-pass filter set at 1–1000 Hz, and filtered data were segmented beginning from 50 ms before the stimulus until 250 ms after the stimulus. The 50-ms period before a stimulus was the baseline. Epochs with responses exceeding ±70 μV were rejected from the analysis, and the remaining data were averaged. In the paired-pulse trial, responses after the second response were obtained by subtracting the single SEP waveform (Fig. 2). PPI by each ISI of 5, 30, and 100 ms (called PPI5, PPI30, and PPI100, respectively) was calculated as the ratio between the response of a single pulse and the second response of the subtracted paired pulse.

### Active joint position matching task

**FIG. 3.**
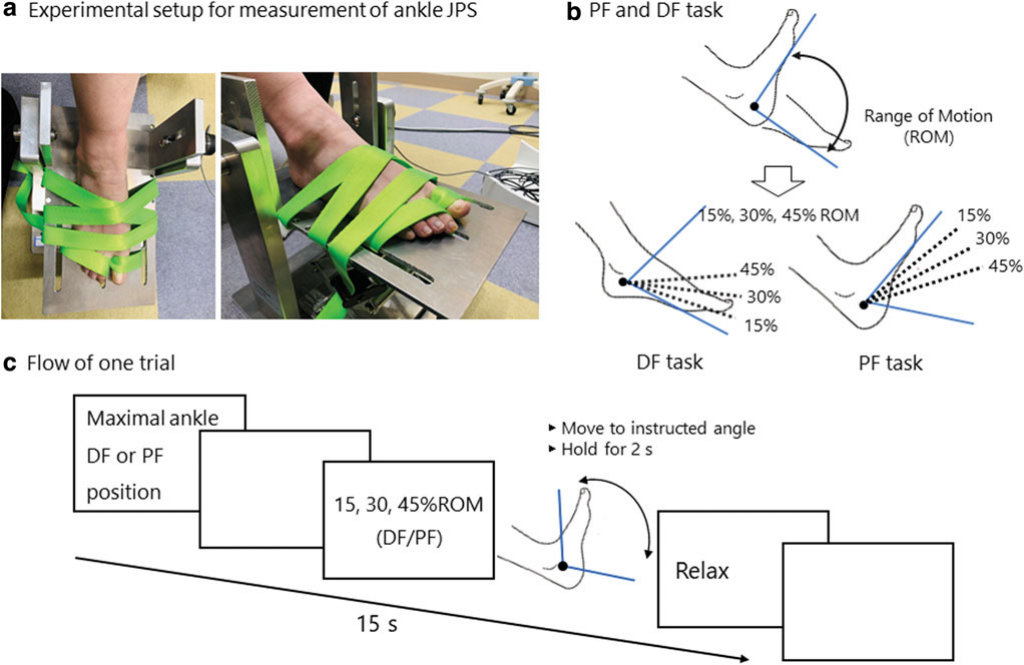
Protocol of active joint position matching task. **(a)** Experimental setup for measurement of ankle JPS. **(b)** Detailed explanation for PF and DF task. Dotted lines mean target angle for each %ROM. **(c)** Flow of one trial. DF, dorsal flexion; JPS, joint position sense; PF, plantar flexion; ROM, range of motion.

The performance of active joint position matching task was assessed as ankle JPS. Each participant sat down on a reclining chair and placed their right foot on the original device (Fig. 3a). This foot was held by a belt on the plate. The knee and hip joints were positioned at 30° and 90° of flexion, respectively. A desk was placed in front of the participant to hide their legs.

Before beginning the task, the range of motion (ROM) of the right ankle joint was measured by the original device. Based on this, the examiner calculated three angles with 15%, 30%, and 45% of ROM (referred hereinafter as 15%ROM, 30%ROM, and 45%ROM, respectively) in each participant. Participants were instructed to memorize these three angles of ankle position from a maximal ankle dorsal or plantar flex position for PF or DF task (Fig. 3b). The examiner moved the participants' foot to each angle in the following order: 15%ROM, 30%ROM, 45%ROM, 15%ROM, 30%ROM, and 45%ROM. The duration for memory was 2 seconds in each position. The examiner then moved the participants' foot to a starting position with a maximal ankle dorsal or plantar flex position for a PF or DF task.

After these memory processes, the examiner instructed participants to focus on the monitor in front of them. We used a custom-built PC program (DASYLab version 2016, dasylab.com) for the task (Fig. 3c). Each trial consisted of 15 seconds from the warning signal to the start of the next trial. Participants were instructed to move to the starting maximal ankle dorsal or plantar flex position after the warning signal. In one trial, a warning signal and blank page were presented for 2 seconds, followed by instructions to adopt a specific ankle plantar or DF angle. Participants were instructed to flex their ankles to the instructed angle, hold this position for 2 seconds, and then relax the ankle. Subsequently, the blank page was presented until the next warning signal. Participants were instructed to stay relaxed, except when performing the task. Each measurement block consisted of three different angles, namely, 15%ROM, 30%ROM, and 45%ROM, from the maximal ankle dorsal or plantar flex position for a PF or DF task. The order was randomized by a computer, and 10 trials of each task were presented per measurement block.

### Menstrual distress questionnaire

To evaluate the prevalence and severity of their menstrual symptoms, each subject filled out the self-reporting MDQ.^35^ In brief, the MDQ consists of 48 symptoms in eight categories: pain, concentration, behavioral change, autonomic reactions, water retention, negative affect, arousal, and control. Participants assessed symptoms by assigning a score of 1 (no symptoms), 2 (minimal), 3 (mild), 4 (moderate), 5 (strong), or 6 (severe) to each item across the eight categories. In each participant, the MDQ was administered three times during follicular, ovulatory, and luteal phases. The sum of scores in each category was used to evaluate each symptom.

### Data analysis and statistics

For behavioral data, the absolute error angle and instructed angle were calculated in each trial. The average absolute error angle from 10 trials was used for behavioral assessment in each type of task (15%ROM, 30%ROM, and 45%ROM). The average absolute error was entered into a three-factor repeated-measures analysis of variance (ANOVA) with “phase” (follicular, ovulatory, and luteal), “task” (DF and PF), and “angle” (15%ROM, 30%ROM, and 45%ROM) as within-subject factors. For neurophysiological data, single-pulse SEP was entered into one-factor repeated-measures ANOVA with “phase” (follicular, ovulatory, and luteal) as a within-subject factor. PPI was entered into a two-factor repeated-measures ANOVA with “phase” and “ISI” (5, 30, and 100 ms) as within-subject factors.

For menstrual distress, MDQ scores were entered into one-factor repeated-measures ANOVA with “phase” as a within-subject factor. The relationships between behavioral, neurophysiological, and menstrual distress assessments were analyzed using Pearson's correlation coefficients.

In all analyses using repeated-measures ANOVA, the Greenhouse–Geisser correction was used to correct for nonsphericity if necessary, and Bonferroni's *post hoc* tests were used for pair-wise comparisons. A *p*-value of <0.05 was considered statistically significant. Data were analyzed using the SPSS version 18 (IBM Corp., Armonk, NY). All data are expressed as mean ± standard error of the mean.

## Results

### Participants

Fourteen right-footed female physical education students (age range, 19–22; age, 20.9 ± 1.2 years; height, 159.2 ± 7.9 cm; weight, 55.01 ± 8.31 kg) participated in this study. During the tracking period, five participants were excluded from the analysis because three of them had irregular menstruation and two could not participate in the measurement because of personal circumstances. All participants had no injuries in the lower extremities for a minimum of 3 months preceding the study. No participant had neurological, rheumatologic, orthopedic, or vestibular dysfunction issues, and none had a history of pregnancy. None used oral contraceptives or other hormone-stimulating medications for the 6 months before the study.

### Single-pulse SEP and PPI

**FIG. 4.**
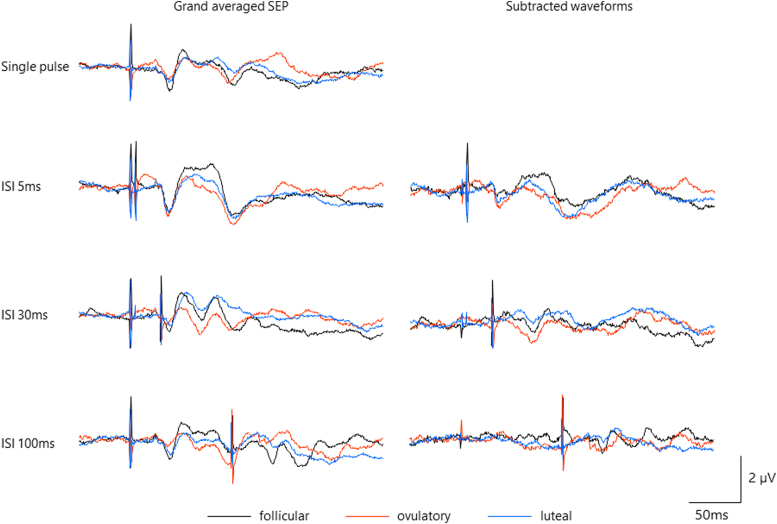
Grand averaged waveforms in all participants. Black, red, and blue lines show grand averaged data in follicular, ovulatory, and luteal phases, respectively.

**FIG. 5.**
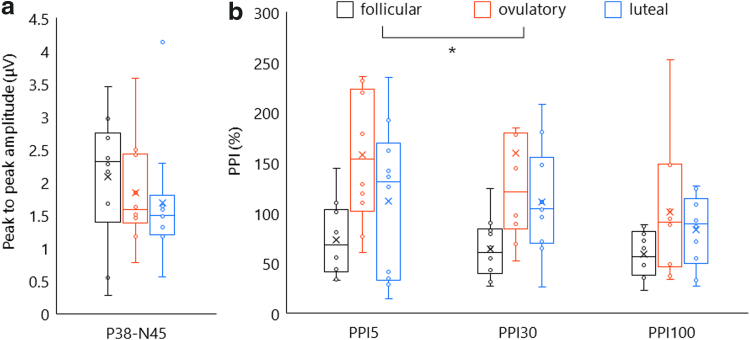
Single-pulse SEP and PPI in each menstrual cycle phase. Black, red, and blue indicate the data in follicular, ovulatory, and luteal phases, respectively. **(a)** Change in single-pulse SEP amplitude. N38-P45 peak-to-peak amplitude did not significantly alter throughout the menstrual cycle. **(b)** Change in PPI with each ISI of 5, 30, and 100 ms. We could find significant increase in PPI in the ovulatory phase compared with the follicular phase, but not dependent on ISI. ISI, interstimulus interval.

For single-pulse SEP, one-factor repeated-measures ANOVA revealed no main effects of “phase” (*F*[2,18] = 0.691, *p* = 0.514, *ηp*^2^ = 0.071). Figures 4 and 5 show grand averaged waveforms and change in PPI throughout the menstrual cycle. Two-factor repeated-measures ANOVA revealed significant main effects of “ISI” (*F*[2,18] = 4.107, *p* = 0.034, *ηp*^2^ = 0.313) and “phase” (*F*[2,18] = 5.476, *p* = 0.014, *ηp*^2^ = 0.378), whereas no significant interaction with “ISI” × “phase” (*F*[4,36] = 0.597, *p* = 0.667, *ηp*^2^ = 0.062). *Post hoc* test showed significantly weaker PPI in the ovulatory phase than in the follicular phase (*p* = 0.048).

### Active joint position matching task

**FIG. 6.**
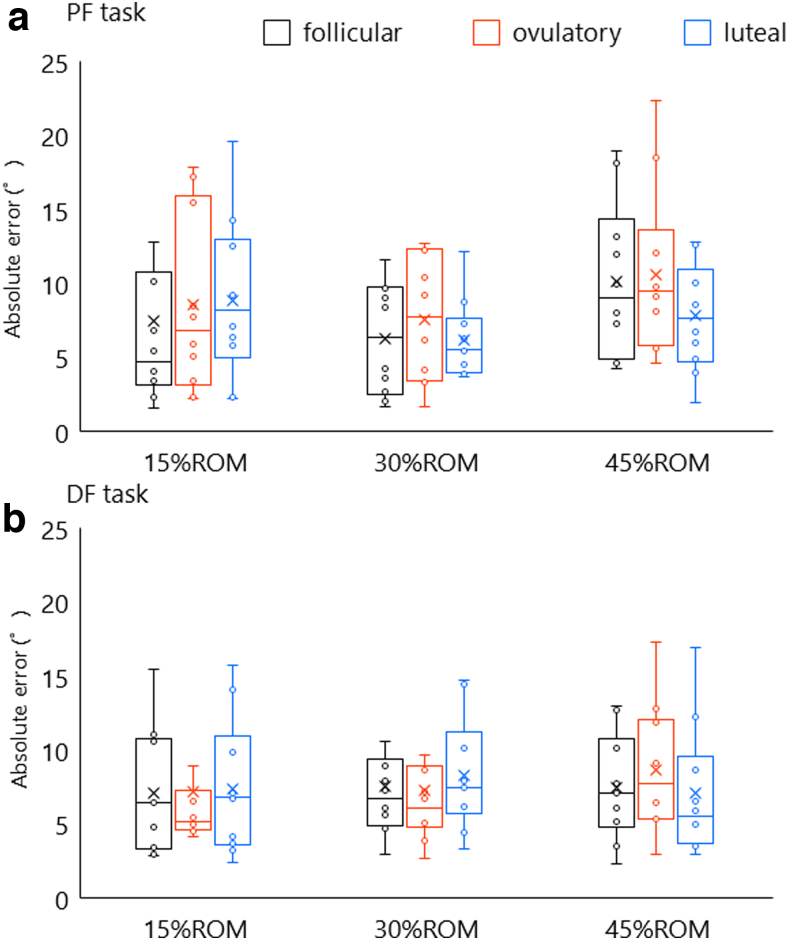
Ankle JPS in each menstrual cycle phase. Black, red, and blue indicate the data in follicular, ovulatory, and luteal phases, respectively. **(a, b)** Performances in PF and DF tasks in each menstrual phase. These data indicated that ankle JPS did not alter throughout the menstrual cycle.

Figure 6 shows the ankle JPS in each menstrual cycle phase. Three-factor repeated-measures ANOVA revealed no significant interaction with “phase” × “task” × “angle” (*F*[2.171,19.543] = 0.706, *p* = 0.491, *ηp*^2^ = 0.078), “phase” × “task” (*F*[2,18] = 0.148, *p* = 0.863, *ηp*^2^ = 0.016), “phase” × “angle” (*F*[1.954,17.583] = 1.083, *p* = 0.359, *ηp*^2^ = 0.107), and “task” × “angle” (*F*[1.278,11.499] = 1.408, *p* = 0.270, *ηp*^2^ = 0.135). No significant main effect of “phase” (*F*[2,18] = 0.336, *p* = 0.719, *ηp*^2^ = 0.036), “task” (*F*[1,9] = 0.561, *p* = 0.473, *ηp*^2^ = 0.059), and “angle” (*F*[1.132,10.185] = 0.797, *p* = 0.408, *ηp*^2^ = 0.081) was found.

### Menstrual distress

**FIG. 7.**
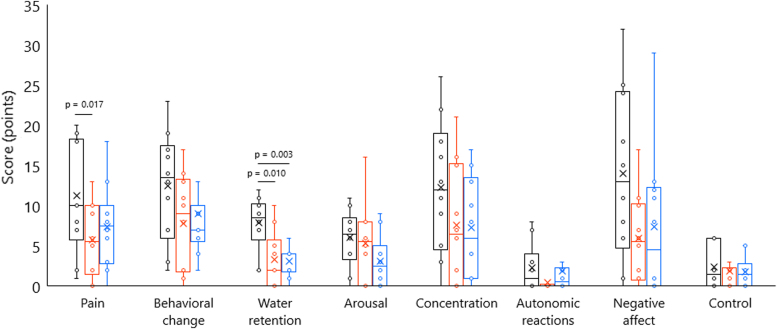
Menstrual distress questionnaire scores in each menstrual cycle. Black, red, and blue indicate the data in follicular, ovulatory, and luteal phases, respectively. In total, menstrual syndromes tend to be worse in the follicular phase; notably, pain was higher than that in the ovulatory phase and water retention was higher than those in ovulatory and luteal phases.

Figure 7 shows the MDQ score in each menstrual cycle phase. Regarding MDQ scores, one-factor repeated-measures ANOVA revealed significant main effects of “phase” in the categories of pain (*F*[2,18] = 8.472, *p* = 0.003, *ηp*^2^ = 0.485) and water retention (*F*[2,18] = 11.724, *p* = 0.001, *ηp*^2^ = 0.566). *Post hoc* tests showed significantly higher MDQ score in the follicular phase than in the ovulatory phase (pain: *p* = 0.017, water retention: *p* = 0.010) and luteal phase (water retention: *p* = 0.003). On the contrary, no significant main effects of “phase” were found in other categories.

### Correlation analysis

Tables 1 and 2 show the results of the correlation analysis between ankle JPS and neurophysiological (Table 1) and emotional (Table 2) measures by each menstrual cycle phase. Regarding relation with single-pulse SEP amplitude and three types of PPI as neurophysiological measures, we found significant correlation of PPI30 and DF task with 15%ROM (*r* = 0.652, *p* = 0.041) and 30%ROM (*r* = 0.910, *p* < 0.001) in the follicular phase. Significant correlation was observed between PPI100 and DF task with 30%ROM (*r* = −0.698, *p* = 0.025) in the luteal phase.

**Table 1.** Relationship Between Ankle Joint Position Sense and Neurophysiological Parameters (Somatosensory Evoked Potential and Paired-Pulse Inhibition)

Task	Phase	Joint angle (%ROM)	P38-N45 pp		PPI5		PPI30		PPI100	
			r	p	r	P	R	p	r	p
DF	Follicular	15	0.479	0.161	−0.401	0.251	0.652^*^	0.041	0.605	0.064
		30	0.442	0.201	−0.438	0.206	0.910^*^	0.000	−0.029	0.937
		45	0.514	0.129	−0.122	0.738	0.582	0.084	−0.425	0.221
	Ovulation	15	0.221	0.540	−0.068	0.852	0.050	0.890	0.129	0.723
		30	0.248	0.489	−0.085	0.816	0.008	0.983	0.022	0.952
		45	−0.270	0.450	0.300	0.400	0.145	0.690	0.306	0.390
	Luteal	15	−0.370	0.293	0.257	0.473	0.034	0.926	−0.449	0.193
		30	0.520	0.124	−0.142	0.696	−0.569	0.086	−0.698^*^	0.025
		45	0.129	0.722	−0.120	0.742	−0.227	0.528	−0.565	0.089
PF	Follicular	15	0.164	0.651	0.029	0.937	−0.381	0.278	0.444	0.199
		30	−0.189	0.600	0.431	0.213	−0.260	0.468	−0.359	0.308
		45	−0.111	0.760	0.258	0.473	−0.021	0.953	−0.426	0.219
	Ovulation	15	0.076	0.835	0.082	0.821	−0.016	0.965	0.414	0.234
		30	0.206	0.568	−0.390	0.265	−0.403	0.249	−0.104	0.775
		45	0.056	0.878	−0.394	0.259	−0.278	0.437	−0.295	0.408
	Luteal	15	−0.071	0.846	0.159	0.660	−0.543	0.105	−0.166	0.646
		30	−0.224	0.534	−0.165	0.649	−0.010	0.979	−0.042	0.909
		45	0.250	0.485	−0.371	0.292	0.124	0.733	0.008	0.983

^*^ Shows significant correlations (*p* < 0.05).

DF, dorsal flexion; PF, plantar flexion; PPI, paired-pulse inhibition; ROM, range of motion.

**Table 2.** Relationship Between Ankle Joint Position Sense and Premenstrual Syndrome

Task	Phase	Joint angle	Pain		Behavioral change		Water retention		Arousal		Concentration		Autonomic reactions		Negative affect		Control	
			r	p	r	p	R	p	r	p	r	p	r	p	r	p	r	p
DF	Follicular	15	0.049	0.892	−0.107	0.769	0.254	0.479	0.443	0.200	0.391	0.264	−0.220	0.541	0.128	0.725	−0.080	0.827
		30	−0.030	0.934	0.136	0.708	0.414	0.234	0.605	0.064	0.259	0.470	−0.382	0.276	0.361	0.305	−0.328	0.354
		45	−0.073	0.840	0.214	0.553	0.035	0.924	0.559	0.093	0.430	0.215	−0.021	0.954	0.275	0.442	−0.186	0.606
	Ovulation	15	−0.354	0.316	−0.153	0.673	−0.292	0.413	−0.358	0.309	−0.275	0.441	−0.103	0.776	−0.288	0.420	−0.129	0.723
		30	−0.145	0.689	−0.043	0.905	−0.347	0.327	−0.216	0.548	−0.296	0.407	0.013	0.971	−0.341	0.335	−0.082	0.821
		45	0.076	0.835	0.292	0.413	−0.040	0.913	0.108	0.766	0.269	0.452	0.349	0.322	0.181	0.617	0.291	0.415
	Luteal	15	0.442	0.201	0.186	0.606	−0.127	0.726	0.490	0.150	0.336	0.343	0.158	0.662	0.256	0.476	0.330	0.352
		30	0.333	0.348	−0.107	0.768	0.017	0.963	0.445	0.197	0.090	0.804	0.072	0.843	0.193	0.594	0.003	0.994
		45	−0.054	0.881	−0.368	0.296	−0.207	0.567	0.312	0.379	−0.078	0.831	−0.179	0.621	0.094	0.797	−0.179	0.620
PF	Follicular	15	0.095	0.795	−0.062	0.865	−0.442	0.201	−0.244	0.497	0.189	0.601	0.341	0.335	−0.252	0.483	0.219	0.543
		30	0.042	0.908	0.247	0.491	−0.192	0.595	0.116	0.750	0.294	0.410	0.177	0.625	−0.116	0.750	0.573	0.084
		45	−0.299	0.402	−0.054	0.882	−0.140	0.699	0.134	0.712	0.073	0.841	−0.256	0.475	−0.313	0.378	0.286	0.423
	Ovulation	15	0.130	0.721	0.226	0.531	0.057	0.876	−0.269	0.452	0.189	0.601	0.270	0.451	0.238	0.509	0.276	0.441
		30	0.238	0.508	−0.036	0.920	0.140	0.699	0.002	0.996	0.420	0.227	0.356	0.312	0.277	0.439	0.366	0.298
		45	−0.101	0.781	0.313	0.378	0.051	0.889	0.114	0.754	0.336	0.343	0.111	0.760	0.140	0.699	0.172	0.634
	Luteal	15	0.431	0.213	0.437	0.207	−0.285	0.425	0.283	0.427	0.464	0.176	0.535	0.111	0.501	0.140	0.416	0.232
		30	0.471	0.170	0.333	0.346	0.097	0.791	0.059	0.871	0.271	0.449	0.151	0.677	0.080	0.827	0.308	0.386
		45	0.449	0.194	−0.114	0.754	0.806^*^	0.005	−0.351	0.320	0.017	0.962	−0.378	0.281	−0.409	0.241	0.375	0.285

^*^ Shows significant correlations (*p* < 0.05).

With respect to the relation with MDQ scores as emotional measures, we showed significant correlation between PF task with 45%ROM and water retention (*r* = 0.806, *p* = 0.005) in the luteal phase.

## Discussion

This study examined whether menstrual cycle phases influence the neural excitability in S1 and JPS using an active joint position matching method to test our hypothesis that PPI will decrease in the ovulatory phase when the estrogen concentration is high and ankle JPS will be altered with accompanying change in PPI. The main findings were as follows: (1) PPI decreased in the ovulatory phase compared with the follicular phase and (2) ankle JPS was not changed throughout the menstrual cycle.

Decreased PPI in the ovulatory phase may be the reason for estrogen altering the neural inhibition and facilitation balance throughout the menstrual cycle. First, it is believed that estrogen enhances the frequency of neuronal firing.^34^ Estradiol administration to ovariectomized rats induced a decrease in the number of inhibitory synaptic inputs, an increase in the number of excitatory synapses, and an enhancement of the frequency of neuronal firing.^34^ Estrogen decreases the firing threshold of a neuron by increasing the activities of *N*-methyl-d-aspartate-mediated glutamate receptor, which, therefore, increases cortical excitability.^36^ Estrogen also binds with the GABA receptor, alters chloride conductance and, therefore, reduces GABA-mediated inhibition.^27^ In fact, by inhibiting GABAergic inhibition, estrogen activates pyramidal neurons.^27^ Although the involvement of GABAergic activity in PPI seems to differ depending on the sensory modality,^28,37,38^ PPI in somatosensory^28^ and auditory^38^ modality was mediated by GABAergic activity. Therefore, we assumed that the elevation of the estrogen level induced a decrease in GABAergic inhibition in the ovulatory phase, which leads to the downregulation of PPI. With regard to auditory PPI, these findings were supported by a human study revealing sex differences in prepulse inhibition using auditory stimuli (auditory PPI). Aasen et al.^39^ reported that women displayed less auditory PPI than men. In addition, supporting evidence comes from observations of similar auditory PPI between men and women in conditions with low estrogen levels^40^ or lower auditory PPI when estrogen levels in women are high.^41^ Based on those findings, the present results might indicate that estrogen decreased PPI using somatosensory modality during the ovulatory phase, which agree with the results of previous studies using auditory PPI. Another possible explanation for decreased PPI during the ovulatory phase would be estrogen-induced dopamine (DA) neural activity. So far, studies have well established that estrogens modulate DA neural activity in the dorsal striatum.^42–44^ Even in humans, direct infusion of estrogen into the dorsal striatum increased DA release,^45,46^ and this enhancing effect of E2 is mediated by E2 receptors and mGlu5 receptors.^46^ Animal and human studies suggest increased striatal D1 and D2 receptor density in periovulatory versus follicular phases in menstruating women.^47,48^ Thus, some evidence supports higher DA neural activity in the periovulatory phase than in the remaining phases of the menstrual cycle. Since there are DA receptors in S1 as well as in subcortical regions and temporal and parietal lobes,^49^ the present results might be attributed to the increased DA neural activity caused by estrogen. Alternatively, sex steroid hormone-mediated changes in the somatosensory threshold at a sensorineural level might alter the sensitivity for the first pulse and thus PPI throughout the menstrual cycle. In other words, a reduction in somatosensory sensitivity would blunt the effectiveness of the first pulse in inhibited amplitude; however, in this study, such a change in single-pulse SEP amplitude was not observed. Therefore, decreased PPI in the ovulatory phase is thought not to be caused by somatosensory sensitivity for the first pulse.

This study showed that ankle JPS using an active joint position matching method did not change throughout the menstrual cycle. The present active joint position matching task reflects proprioception, including external sensory feedback and sensorimotor integration^20^ for repositioning to the specific joint displacement. Fridén et al.^50^ have demonstrated proprioceptive defects during menstruation in consistence with other studies.^22,23^ These results were explained by the fluctuation of the hormonal level related to the increased joint laxity and negative emotional aspects (*i.e.*, premenstrual syndrome). One possible explanation for this dissimilarity with the present results would be the difference in the measured joint. Many studies pointed out an increase in knee joint laxity during ovulatory and luteal phases,^19,51,52^ confirming another previous result showing increases in joint laxity across hormonal fluctuation.^53^ Compared with the knee joint laxity, ankle laxity did not fluctuate throughout the menstrual cycle.^19,54^ Therefore, proprioception in the ankle joint seems not to change throughout the menstrual cycle because there was no change in the ankle joint laxity, unlike in the knee joint. For the negative emotional aspects, since Fouladi et al.^23^ raised proposed emotional condition as another reason for the worse JPS during the follicular phase, we could not rule out this aspect. In truth, we could find worse emotional condition in pain and water retention during the follicular phase. Even if we had examined the correlation between ankle JPS and emotional condition, we could not find significant correlation between them, except for one result (PF task with 45%ROM and water retention in the luteal phase). Based on these results, negative emotional aspects related to the menstrual cycle seem not to influence the present ankle JPS. Alternatively, Aydoğ et al.^22^ reported that cortical activity such as peripheral stimulation-evoked potentials could possibly be a reason for the deficits in JPS in the follicular phase, although it was not clear how female hormones affect proprioception. Besides, proprioceptive sensory threshold evaluated by vibration stimuli increases during the follicular phase even if this did not directly reflect cortical activity.^55,56^ As the present findings result from single- and paired-pulse evoked potential throughout the menstrual cycle, we could show deterioration in the inhibitory function during the ovulatory phase compared with that during the follicular phase. However, considering the absence of changes in ankle JPS when analyzed using an active joint position matching method throughout the menstrual cycle, these neurophysiological alterations would be likely to have a limited effect on proprioception in the ankle joint.

We found some symptoms such as pain, water retention, and negative affect in the follicular phase compared with the ovulatory and luteal phases as a result of higher MDQ scores. These symptoms generally appear during the luteal phase and disappear after the onset of menstruation, but individual difference occur on the basis of timing and severity. In fact, previous studies reported that the appearance and severity of menstrual symptoms were higher in the follicular phase than in the luteal phase.^57,58^ It appeared that many participants felt some menstrual symptoms during the follicular phase similar to these previous studies. Although this study showed one significant correlation between PF task with 45%ROM and water retention in the luteal phase than MDQ alone, previous studies have reported some correlation between menstrual symptoms and cognitive and sensory functions.^59,60^ To identify the influence of menstrual symptoms on PPI with somatosensory modality and ankle JPS using an active joint position matching method, further studies are needed.

This study has several limitations with respect to clarifying menstrual cycle-dependent differences in PPI with somatosensory modality and ankle JPS. First, the sample size was small. In this study, we recruited 14 women who provided informed consent, but 4 of them could not finish all experiments for several reasons. To obtain robust evidence, we should retest the present results in many participants. Second, we could not ascertain fluctuations in sex steroid hormone concentration because regular menstrual cycle length and changes in basal body temperature were used to determine the menstrual cycle phases. We adopted these methodologies to determine each phase since previous studies identified each phase based on the change in basal body temperature.^22,61^ However, Metcalf, Skidmore, Lowry, and Mackenzie^62^ reported that only 62% of women aged 20–25 years ovulated in all cycles tested and anovulatory cycles were even more common in younger women. Therefore, it is essential to estimate fluctuation in sex steroid hormonal concentration for accurate identification of menstrual cycle phases. As this is a pilot study, further studies that directly assess sex steroid hormones are needed to clarify menstrual cycle-dependent changes in PPI with somatosensory modality and ankle JPS using an active joint position matching method.

In conclusion, the present results suggested that the phases of the menstrual cycle affect neural excitability in S1 as shown in the decreased PPI in the ovulatory phase. On the contrary, ankle JPS analyzed using the active joint position matching method did not change throughout the menstrual cycle.

## Abbreviations Used

ANOVA: analysis of variance
AS: ankle sprain
DA: dopamine
DF: dorsal flexion
EEG: electroencephalogram
ES: electrical stimulation
GABA: gamma-aminobutyric acid
ISI: interstimulus interval
JPS: joint position sense
MDQ: menstrual distress questionnaire
PF: plantar flexion
PPI: paired-pulse inhibition
ROM: range of motion
S1: somatosensory cortex
SEP: somatosensory evoked potential
